# Polyphenol enriched ethanolic extract of *Cajanus scarabaeoides* (L.) Thouars exerts potential antifilarial activity by inducing oxidative stress and programmed cell death

**DOI:** 10.1371/journal.pone.0208201

**Published:** 2018-12-06

**Authors:** Anindya Sundar Ray, Nikhilesh Joardar, Suprabhat Mukherjee, Chowdhury Habibur Rahaman, Santi P. Sinha Babu

**Affiliations:** 1 Ethnopharmacology Laboratory, Department of Botany, Visva-Bharati University, Santiniketan, India; 2 Parasitology Laboratory, Department of Zoology, Visva-Bharati University, Santiniketan, India; State University of Ponta Grossa, BRAZIL

## Abstract

Development of antifilarial drug from the natural sources is considered as one of the most efficacious, safe, and affordable approaches. In this study, we report the antifilarial activity of a leguminous plant *Cajanus scarabaeoides* (L.) Thouars. The polyphenol-rich ethanolic extract obtained from the stem part of the plant *C*. *scarabaeoides* (EECs) was found to be efficient in killing the filarial nematode *Setaria cervi* in all the three developmental stages viz. oocytes, microfilariae (Mf) and adults with LD_50_ values of 2.5, 10 and 35 μg/ml, respectively. While studying the molecular mechanism of action, we found that induction of oxidative stress plays the key role in inducing the mortality in *S*. *cervi*. The redox imbalance finally results in activation of the nematode CED pathway that executes the death of the parasite. Intriguingly, EECs was found to be selectively active against the worm and absolutely non-toxic to the mammalian cells and tissues. Taken together, our experimental data demonstrate that *C*. *scarabaeoides* can be chosen as an affordable natural therapeutic for treating filarial infection in the future with high efficacy and less toxicity.

## Introduction

Lymphatic filariasis (LF) is known to be a potential threat to the people residing in the tropics and subtropics. It is still posing its superiority over decades [1]. 856 million people in 52 countries are threatened by lymphatic filariasis and require potent therapeutics [1]. *Wuchereria bancrofti*, *Brugia malayi*, and *Brugia timori* are known filarial parasites responsible for LF in human beings [1]. At present, the available chemotherapies include diethylcarbamazine citrate, albendazole, and ivermectin, but their efficacy is limited to microfilarial stage only [2]. Moreover, resistance to such synthetic drugs is also a serious concern [3]. However, WHO has taken a resolution to eliminate LF through the Global Programme to Eliminate Lymphatic Filariasis (GPELF) by 2020 [4]. It includes strategies to combat LF through mass drug administration (MDA), morbidity management and vector control [1]. At present, potent adulticidal formulations are in high demand. Although a lot of synthetic and natural compounds [5], effective in killing the parasites have evolved, still it is necessary to explore more efficacious botanicals with ethnopharmacological properties [6].

A very small percentage of total plant species in the world has so far been investigated for their bioactivities. Only about 6% of total higher plant species (angiosperms and gymnosperms) have been screened for biological activity and among them, nearly 15% have been evaluated for their phytochemical efficacy [7]. As a result, there remains a vast opportunity to work with natural products for ameliorating the disease.

*Cajanus scarabaeoides*, commonly known as Ban kurti is a twinning herb of family Leguminosae. Different parts of *C*. *scarabaeoides* have a wide range of ethnomedicinal uses for treatment of anemia, smallpox, gonnorhoea, rinderpest, sores, dysentery, cholera, swelling and different inflammatory disorders [8–12]. The pharmacognostic characterization of the plant *C*. *scarabaeoides* has been described previously in a study by Ray and Rahaman with an objective to highlight its qualitative and quantitative estimation of different phytochemical groups [8]. Despite its varied utilities in traditional medicine antifilarial potential of this plant has not been elucidated earlier. The present study aims to demonstrate the *in vitro* antifilarial potential of the polyphenol-enriched optimized ethanolic extract of the stem part of *Cajanus scarabaeoides* (L.) Thouars plant (EECs). This study is a maiden report on the efficacy of the polyphenol-rich ethanolic extract of the plant *C*. *scarabaeoides* as a novel natural nontoxic antifilarial agent.

## Materials and methods

### Chemicals and reagents

Chief purity grade solvents were purchased from Merck, India and Milli-Q water (Milli-Q Academic with 0.22μm Millipak R-40) was used. RPMI-1640, DMEM (Hi-Media, India), fetal bovine serum (Gibco, USA), MTT (Merck, India), NBT (Merck, India), Hoechst (Sigma, USA), Ethidium bromide (EtBr) and Acridine orange (AO) (Merck, India), DMSO (Merck, India), N-acetyl-l-cysteine (NAC) and 2´,7´-dichlorofluorescein diacetate (H_2_DCFDA) (Sigma, USA), Z-VAD-FMK obtained from Cayman Chemicals (USA), ethanol, chloroform, ethyl acetate, petroleum ether (Merck, India) were used for this study.

### Plant material

Freshly collected root, stem, leaf, and fruit parts of *Cajanus scarabaeoides* (L.) Thouars (Leguminosae) harvested from Muluk (N 23°38'30.3", E 087° 42'29.2"), Sian block, Birbhum district (23.8402° N, 87.6186° E), West Bengal, were used for the study. The plant was identified following Ray and Rahaman [8], and authenticated by an expert botanist and deposited in the herbarium of the Department of Botany, Visva-Bharati University (ANSHS/Bot/01/2018). The plant sample collection study was conducted following the guidelines of IUCN (International Union for Conservation of Nature).

### Preparation of extracts and its partial characterization

The collected plant parts of *C*. *scarabaeoides* were washed and air-dried under a shed at room temperature. Dried plant samples were grounded. Powdered plant samples were separately extracted with different organic solvents such as aqueous ethanol, chloroform, ethyl acetate, and petroleum ether. Each extraction was performed by adding the extraction solvent at 1:10 w/v ratio and the extraction was conducted for 24 h at room temperature with continuous stirring. After extraction, the extract was filtered using a 0.2μM filter and the filtrate was collected as the crude extract. The crude extract was air dried and dissolved in dimethyl sulfoxide (DMSO). Each solvent extract was then tested on the filarial parasite *Setaria cervi* (Rudolphi) for their lethal action. Out of all four solvent extracts tested, ethanolic stem extract was found to be the most active. Later the extract was characterized by HPTLC analysis following Mukherjee et al. [13]. For HPTLC analysis the extract was dissolved in methanol (100μg/ml) and separated in thin layer silica gel 60 F_254_ immobilized on aluminum plate (10×10 cm; Merck, Germany) using three different solvent systems. Solvent system 1; comprised of toluene: ethyl acetate: formic acid (5:4:1); solvent system 2; comprised of toluene: ethyl acetate: formic acid (4.5:3:0.2), and solvent system 3; composed of ethyl acetate: acetic acid: formic acid: water (10:1.1:1.1:2.5). The separations were conducted in Twin Trough chamber (20×10 cm) previously equilibrated with the mobile phase solvents (10 mins at 25°C). Similarly standard polyphenols (gallic acid, caffeic acid, ferulic acid, resveratrol) and flavonoids (rutin, quercetin, catechin) (5μg each) were separated using the same solvent systems. Samples were applied on the TLC plate using Camag Linomat V applicator (Switzerland) fitted with a microsyringe. After separation, plates were dried and scanned by a TLC scanner (Camag TLC scanner 3) equipped with UV lamp. Reference compounds were determined in the extract by means of retention factor (R_f_) and the peak attributes (height, area, and concentration) were calculated by linear regression analysis.

**Fig 1:**
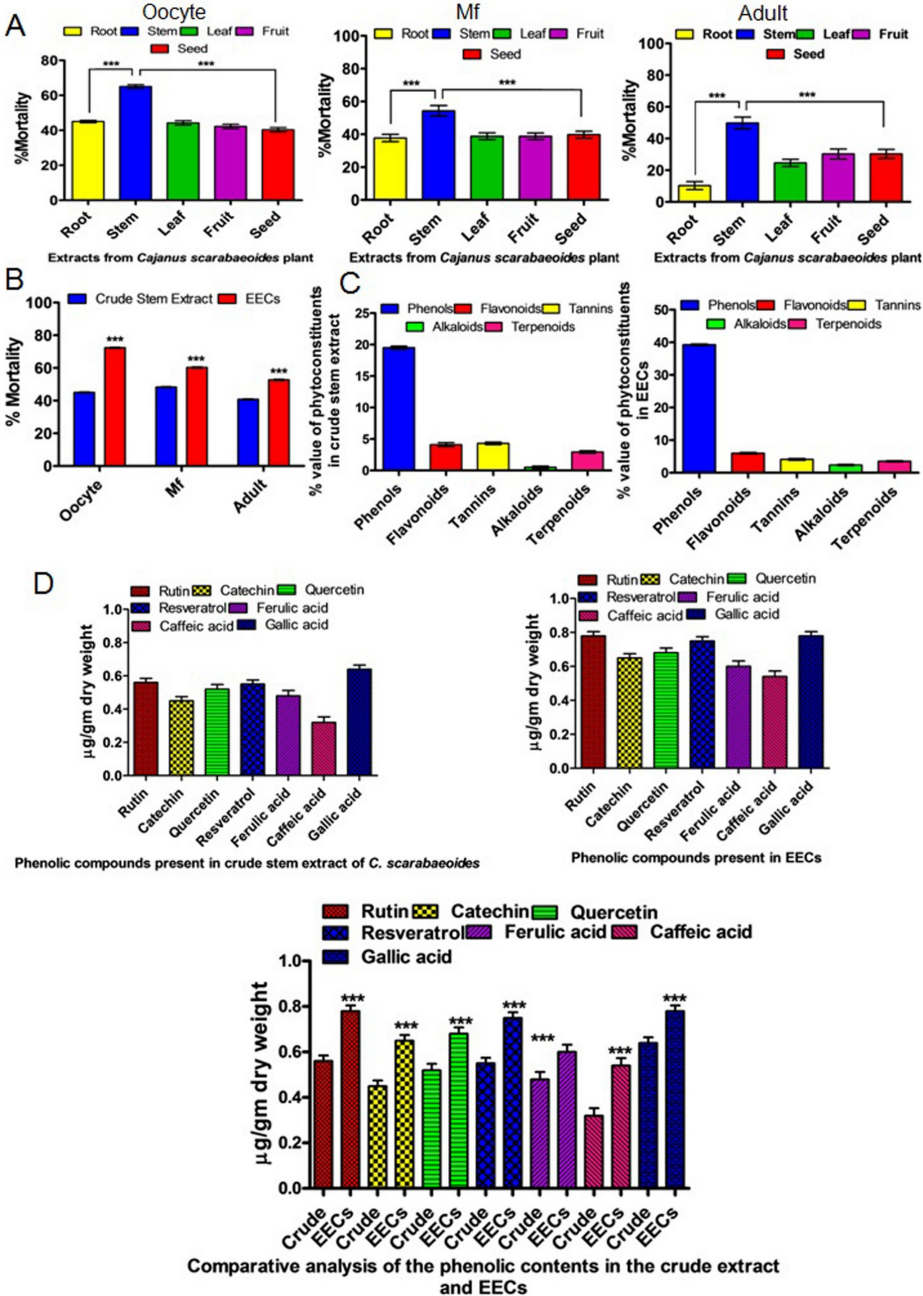
Determination of antifilarial activity in different parts of the plant *Cajanus scarabaeoides* and HPTLC based chemo-profiling of the ethanolic extract of *C*. *scarabaeoides*. A. Efficacy of ethanolic extracts prepared from the root, stem, leaf, fruit, and the seed of *C*. *scarabaeoides* on the different developmental stages (oocytes, Mf and adults) of *S*. *cervi*. B. Determination of the antifilarial potential of the active fraction i.e. the stem extract. C. Presence of different bioactive phytochemicals in the ethanolic extract of *C*. *scarabaeoides*. D. HPTLC based quantification of the different bioactive phytochemicals in the ethanolic stem extract of *C*. *scarabaeoides* prepared under optimum conditions favorable for enriching the polyphenols. Each data is obtained from experiments conducted in triplicate and repeated for three times.

The HPTLC analysis primarily suggested about the abundance of polyphenols (Fig 1, S1 and S2 Figs) and therefore the polyphenol content was optimized in the extract following the method depicted in Mukherjee et al. [14]. Extractions were conducted using the statistically optimized extraction module comprising of 15 ml of 75% ethanol kept at 40°C and extraction time of 120 min. The yield of bioactive polyphenols was determined by measuring the total polyphenol content following Mukherjee et al. [15]. Enrichment of the extract with polyphenols was also investigated by HPTLC analysis using the solvent system toluene: ethyl acetate: formic acid (5:4:1) specific for the polyphenols [13, 16].

Finally, the polyphenol-enriched ethanolic extract was air dried and termed as ethanolic extract of *C*. *scarabaeoides* (EECs). Desired treatment concentrations were prepared by dissolving the dried extract in DMSO and kept in 4°C until further use.

### Antiparasitic activity

#### Filarial parasite

*Setaria cervi*, a bovine filarial nematode, has been used in this study for the evaluation of the antifilarial potential of the plant extracts. The *S*. *cervi*, a WHO-recommended model for filarial research, resembles the periodicity and antigenic pattern of the human filarid [15,17–19]. Motile adults of the parasite *S*. *cervi* were obtained from the abdominal cavity of a freshly slaughtered cow (*Bovis indica* Linn.) from the government-approved local abattoirs and brought into the laboratory in normal saline (0.85%). Then the collected parasites were washed repeatedly with 0.85% saline to avoid any extraneous materials and kept in RPMI-1640 media at 37°C with 5% CO_2_ until further use. Oocytes and Mf were collected from gravid females and kept in the same conditions as an adult.

#### Viability tests (IC_50_, LD_50_ values)

Treatment of the different developmental stages of the filarial parasite *S*. *cervi* with EECs was conducted *in vitro*. *S*. *cervi* gravid females were incubated in 10 ml of complete medium (RPMI supplemented with 10% FBS) as control set, and complete medium in combination with the polyphenol-rich ethanolic extract (EECs) at 25, 50, and 100 μg/ml concentrations in 60 mm sterile petri plates (Tarson, India) as treatment groups. Furthermore, approximately 1.0 × 10^5^ oocytes were incubated in 2 ml of complete medium alone as a control set and with EECs at doses of 2.5, 5, and 10μg/ml as treatment sets. 1.0 × 10^4^ Mf were incubated in complete medium with 10, 25, and 50μg/ml doses of EECs, and without EECs as a control group in 24-well plates (Tarson, India). Adult and Mf cultures were maintained for both 24 h and 48 h at 37°C in a humidified atmosphere of 5% CO_2_ [5, 20]. 1% DMSO was kept as positive control. The entire experiments were repeated thrice in duplicate (adult) and triplicate (oocytes and Mf) manner.

#### MTT assay

The viability of parasites (Mf and adult) was assessed by the MTT [3-(4, 5-dimethyl–thiazol-2-yl)-2, 5-diphenyl-tetra-zolium bromide] reduction assay as described previously by Nayak et al. [21] at the cellular level. This MTT assay was designed to calculate the IC_50_ (the particular concentration in which 50% of the parasites were inhibited, i.e. they are either dead or have seized their movement (partially or permanently)) and LD_50_ (the particular dose in which 50% of the parasites died) values. The IC_50_ value was further used for determining the selectivity index (SI). The assays were carried out in triplicate and repeated at least three times. LD_50_ values were determined by using Origin Pro 6.1.

#### Morphological alterations in the EECs-treated adult parasites

The control and EECs-exposed adult parasites were taken out from the culture media and washed thoroughly in PBS. The intact parasites were fixed in 4% paraformaldehyde overnight. After complete fixation, dehydration in graded alcohol was done and finally embedded in molten paraffin. 5μm of adult parasite tissue sections were prepared for hematoxylin-eosin staining [18, 22].

### Determination of oxidative stress in the filarial parasite

#### Determination of reactive oxygen species (ROS)

Level of lipid peroxidation was assayed by determining the level of malondialdehyde (MDA) through the thiobarbituric acid reactive substance method depicted in Mukherjee et al. [13]. In brief, samples, deproteinized with trichloroacetic acid were dissolved in HCl. Thereafter, thiobarbituric acid was added and boiled for 10 mins in a water bath. Samples were allowed to cool at room temperature. Supernatants were collected from the samples after centrifugation at 10,000×g for 15 mins and the color intensity was measured at 532 nm using a dual-beam spectrophotometer (Shimadzu, Japan). The concentrations of MDA in the test samples were calculated by the following equation:

Malondialdehyde concentration (M) = Absorbance at 532nm/ε and expressed as μmol/mg protein. (ε = Extinction coefficient 1.56 x 10^5^)

Colorimetric NBT assay was performed to measure the altered level of ROS in oocytes, Mf and adults of *S*. *cervi* treated with the extracts following the method of Gucchait et al. [5]. In brief, the control and the treated parasites were incubated in 2% NBT, prepared in PBS for 1 hr at room temperature. Thereafter, the samples were washed with PBS and fixed in methanol. The consequential dark formazan crystals were then dissolved in a mixture of 240 μl of 2 mol/L KOH and 480 μl of DMSO. The absorbance was recorded at 620 nm using a microplate reader (Bio-Rad, USA).

Total intracellular ROS production in the exposed parasites was measured from the parasite lysates with H_2_DCFDA. In brief, the control and the treated parasites were incubated in 0.0004 mol/L H_2_DCFDA for 15 min in the dark. The parasites were washed in PBS and lysed using cell lysis buffer (Cell Lytic) and the absorbance of the supernatants was measured using a spectrofluorimeter (LS55, Perkin Elmer) [23, 24] with an excitation wavelength of 504 nm and an emission wavelength of 529 nm. Each experiment was performed in triplicate and repeated five times.

H_2_O_2_ in the control and treated *S*. *cervi* homogenates was estimated according to Mukherjee et al. [20] with modifications. In brief, 50 μl of parasite homogenate was mixed with 450 μl of distilled water and finally, 1ml dichromate/acetic acid solution (5% potassium dichromate in glacial acetic acid 1:3 v/v) was added to it. The mixture was incubated in a water bath for 10min and subsequently brought to room temperature. The absorbance was measured at 570 nm.

#### Determination of enzymatic and nonenzymatic antioxidant parameters

Control and the EECs treated *S*. *cervi* adult parasites were homogenized in phosphate buffered saline (PBS; 100mM, pH 7.0) (0.2 mol/L monobasic sodium phosphate, 0.2 mol/L dibasic sodium phosphate), centrifuged at 10,000×g for 20 mins at 4°C and the clear supernatant was used for the enzyme assay experiments. Levels of the enzymatic antioxidants such as glutathione S-transferase, superoxide dismutase, catalase, glutathione reductase, and nonenzymatic antioxidant i.e. glutathione were estimated following the methods depicted in our earlier reports [15, 16, 20, 25, 26, 27].

Reduced glutathione (GSH) was estimated following Mukherjee et al. [15, 27]. 150 μl of lysate from control and the treated parasites was mixed with 5% perchloric acid. The mixture was centrifuged at 1000×g for 10 min at 4°C and the supernatants were collected. 100 μl of the supernatant was mixed to a solution of 1.88 ml of 0.1M potassium phosphate buffer (6 ml of 1mol/L KH_2_PO_4_, 94 ml of 1 mol/L K_2_HPO_4_ and the total volume was adjusted to 1lit) (pH 8.0) and 20 μl of DTNB (5,5-dithio-bis-(2-nitrobenzoic acid). The entire mixture was incubated for 3 min at 25°C, the absorbance was measured at 412 nm using a spectrophotometer (Shimadzu 1601 UV-Vis spectrophotometer). Level of GSH was expressed in nanomole/mg protein.

The glutathione S-transferase (GST) activity was determined in the cell-free supernatant following method of Mukherjee et al.[27]. In 1 ml of assay cocktail comprising 980 μl of PBS (pH 6.5), 0.1 mol/L CDNB (1-chloro-2,4-dinitrobenzene) and 0.1 mol/L GSH, 100 μl of cell-free supernatant was added and incubated at 30°C for 5 min. The absorbance was measured at 340 nm. Enzyme activity was expressed as U/mg protein.

Catalase activity was assayed following the method described in Mukherjee et al. [15, 27]. 40 μl of the cell-free supernatant obtained from the parasite homogenate was added to the assay mixture containing 3 ml of H_2_O_2_-phosphate buffer (0.002 mol/L H_2_O_2_ prepared in 0.067 M PBS (pH 7.0) (0.1mol/L Na_2_HPO_4_ and 0.1mol/L KH_2_PO_4_). After vigorous mixing, the absorbance was measured at 240 nm using a UV-VIS spectrophotometer (Shimadzu, Japan). The specific activity of the enzyme was expressed in U/mg protein.

Superoxide dismutase (SOD) activity was determined in the parasite homogenate using the SOD assay kit (Cayman Chemical, USA) following the manufacturer’s guidelines. The intensity of the formazan dye produced by the action of SOD was recorded spectrophotometrically at 495 nm. The enzyme activity was expressed as U/mg protein.

### Determination of apoptosis in the filarial parasite

#### Hoechst 33342 staining

Chromatin condensation in the treated oocytes and Mf was determined microscopically by Hoechst 33342 following Gucchait et al. [5] and subsequently photographed under a fluorescence microscope (Leica, Germany) at 490 nm excitation.

#### Acridine orange (AO)/ ethidium bromide (EtBr) double staining

The apoptogenic property of EECs in the exposed *S*. *cervi* oocytes and Mf was determined using acridine orange (AO)/ethidium bromide (EtBr) following the method of Roy et al. [28] and Gucchait et al. [5] and micrographed under a fluorescence microscope (Dewinter, Italy, blue filter excitation, 460–490 nm).

#### Annexin V-FITC and PI staining

*S*. *cervi* oocytes, collected from the gravid females were treated with 2.5μg/ml and 5μg/ml of EECs and further processed for Annexin V-FITC and PI staining. The Annexin V- FITC and PI staining of oocytes were carried out following Gucchait et al. [5] and micrographed under an inverted fluorescence microscope (Leica, Germany).

#### Immunoblotting

The apoptotic potential of EECs was evaluated through Western blotting. In brief, 70 μg of adult *S*. *cervi* worm lysate treated with EECs was resolved in 12.5% SDS-PAGE and thereafter electrotransferred to PVDF membrane, and incubated overnight with nematode-specific pro-apoptotic EGL-1, CED-4 antibodies (Santa Cruz Biotechnologies, USA) and anti-apoptotic CED-9 antibody (Santa Cruz Biotechnologies, USA) [5, 15, 19]. Further, the primary antibody probed PVDF membranes were incubated with alkaline phosphatase conjugated secondary IgG (Sigma-Aldrich, MO, USA) for at least 6 h. Finally, the protein expressions were visualized using BCIP/NBT and photographed using chemidoc (Bio-Rad, USA).

#### Assay of caspase 3 activity

Caspase activity in the worm homogenate both Mf and adults were determined using the microplate-based Caspase Assay System (Promega, USA) following the manufacturer's guidelines. *p*-nitroaniline labeled DEVD peptide was used as a substrate while Z-VAD-FMK was used as an inhibitor [13].

### Toxicity analyses

#### Assessment of the toxicity of EECs *in vitro* and determination of selectivity index

Cytotoxicity of EECs was investigated using peritoneal macrophages of Wistar rat [27]. Cells were seeded into a 24 well culture plate in complete DMEM and exposed to different concentrations of each solvent extract, containing 1% DMSO as a control. Cells were incubated at 37°C for 24 h in a humidified, 5% CO_2_ environment. The safety of extracts was determined in terms of the selectivity index (SI) which was defined as CC_50_ / IC_50_ (SI = CC_50_/ IC_50_, where SI >1 = more toxic to parasites than to Mϕ, SI <1 = more toxic to Mϕ than to parasites) [29].

#### *In vivo* toxicity

The toxic effects of the active extract i.e. EECs were investigated in Wister rat (*Rattus norvegicus*) model. The preclinical toxicity schedule was comprised of seven days of continuous oral administration of EECs (100, 200 and 500 μg/kg body weight) dissolved in distilled water. After completion of treatment schedule rats were euthanized and sacrificed. Blood was collected through heart puncture and stored with EDTA for further hematological analysis. On the other side, blood collected without EDTA was used for collecting the serum and hepatic biomarkers were assayed accordingly following Mukherjee et al. [13]. The liver was perfused with PBS, chopped in small pieces (5×5 mm), fixed in Bouin’s fixative and processed for histological preparation following Mukherjee at al. [13] and Chowdhury et al. [19]. After histological preparations liver tissue sections were stained with hematoxylin and eosin and observed under brightfield optical microscope (Dewinter, Italy). All animal-related works were performed under the strict supervision of the Institutional Ethical Committee cited below and OECD guidelines.

#### Ethical clearance for the study

The protocol for this study was approved by the Institutional Animal Ethical Committee, Visva-Bharati, Santiniketan– 731 235, West Bengal, India and experiments with small laboratory animals were performed as per the guidelines of Committee for the Purpose of Control and Supervision of Experiments of Animals (CPCSEA); Govt. of India (1819/GO/Ere/S/15/CPCSEA).

## Results

### EECs is enriched with polyphenolic compounds

Our study seeks to investigate the natural antifilarial agent that will have desired efficacy by means of killing the filarial parasites in all the developmental stages and will be affordable to all. Herein, we found that ethanolic stem extract of *C*. *scarabaeoides* possesses significant lethal action against the parasite in all the developmental stages at a considerably low dose (Tables 1 and 2). Whilst studying the possible chemo-profiling in the active ethanolic extract of *C*. *scarabaeoides*, we found an abundance of polyphenolic compounds such as gallic acid, caffeic acid, ferulic acid and flavonoids like catechin, rutin, quercetin etc. (Fig 1). Therefore, phenolic compounds could be the bioactive mediator behind eliciting the antifilarial action of *C*. *scarabaeoides*. So, it is expected that antifilarial activity of the crude extract can be improved if the total polyphenolic content is optimized. This has prompted us to apply the statistical design- based optimization strategy as previously reported by Mukherjee et al. [14–16] for maximizing the yield of the polyphenol in the crude extract. After performing the extraction, we found almost 6.5 folds increase in the polyphenolic content. Moreover, the bioactivity (in terms of inducing % mortality) was found to be increased up to 4 folds (Fig 1). The reason behind such improvement is that the optimized extraction module provides the best possible combinations that simultaneously satisfy high yield and abundance of functionally active polyphenols.

**Table 1:** Evaluation of antifilarial activity in different solvent extracts (crude) of *Cajanus scarabaeoides*.

Plant part^#^	Extraction medium	LD_50_ (μg/ml)			IC_50_ (μg/ml)			CC_50_ (μg/ml)			Selectivity Index		
		O	M	A	O	M	A	O	M	A	O	M	A
Root	Ethanol	6.75±0.34	22.75±0.276	56.75±0.25	4.57±0.65	18.5±0.389	49.7±0.254	5.65±0.254	20.2±0.265	51.65±0.198	1.23±0.02	1.09±0.034	1.03±0.029
	Chloroform	54.25±0.038	69.28±0.045	89.25±0.065	51.25±0.034	62.67±0.054	85.23±0.045	51.67±0.034	63.29±0.056	86.2±0.076	1.00±0.02	1.01±.031	1.01±0.054
	Ethyl acetate	53.25±0.076	62.65±0.054	82.28±0.0123	51.56±0.047	65.28±0.065	86.28±0.0654	51.98±0.045	65.32±0.063	86.4±0.29	1.00±0.043	1.00±0.026	1.001±0.039
	Petroleum ether	48.32±0.043	52.65±0.054	62.67±0.043	45.23±0.054	49.25±0.065	61.25±0.065	45±0.043	49.298±0.054	62.35±0.076	1.00±0.065	1.00±0.543	1.0012±0.0064
**Stem**	Ethanol **(Selected)**	**4±.02**	**15± .264**	**45±0.034**	**2.25±0.0539**	**12.8±0.035**	**35.34±0.032**	**3.5±0.387**	**19.75±0.236**	**42.65±0.167**	**1.5±0.26**	**1.54±0.25**	**1.2±0.17**
	Chloroform	12.23±0.025	21.26±0.05	52.95±0.267	10.25±0.26	18.26±0.02	49.75±0.025	10.29±0.054	18.25±0.025	50.1±0.23	1.003±0.05	0.99±0.24	1.01±0.456
	Ethyl acetate	28.24±0.024	37.67±0.025	49.97±0.25	20.16±0.27	30.25±0.17	43.28±0.268	20.23±0.28	30.278±0.168	43.16±0.025	1.00±0.25	1.00±0.254	0.997±0.26
	Petroleum ether	30.45±0.24	44.26±0.267	50.25±0.38	28.27±0.23	41.26±0.87	48.23±0.24	28.98±0.34	41.78±0.761	49.23±0.26	1.00±0.25	1.00±0.024	1.00±0.28
Leaf	Ethanol	26.75±0.342	42.75±0.2763	58.75±0.258	24.57±0.685	38.5±0.39	49.789±0.25	25.65±0.24	39.2±0.265	50.67±0.18	1.04±0.024	1.01±0.034	1.01±0.029
	Chloroform	64.25±0.38	79.28±0.05	109.25±0.65	59.5±0.03	74.67±0.54	98.23±0.04	59.67±0.34	74.29±0.56	100.2±0.074	1.00±0.045	0.994±.03	1.02±0.54
	Ethyl acetate	63.25±0.76	72.65±0.04	92.28±0.23	68.56±0.047	65.28±0.65	90.28±0.6	68.98±0.45	65.32±0.63	90.4±0.21	1.00±0.43	1.00±0.2	1.0±0.03
	Petroleum ether	68.32±0.4	72.5±0.54	82.67±0.3	65.23±0.54	69.25±0.65	75.25±0.5	65±0.04	69.9±0.54	74.35±0.7	1.00±0.5	1.00±0.42	0.99±0.6
Fruit	Ethanol	29.85±0.2	52.5±0.2	68.75±0.8	22.57±0.6	48.5±0.3	59.9±0.2	25.65±0.24	49.2±0.265	59.95±0.8	1.13±0.24	1.01±0.04	1.0±0.09
	Chloroform	65.25±0.38	78.8±0.05	99.2±0.6	62.5±0.3	75.6±0.5	96.23±0.4	69.67±0.34	70.29±0.5	98.2±0.16	1.11±0.5	0.994±.03	0.97±0.4
	Ethyl acetate	63.5±0.7	78.6±0.04	98.28±0.3	75.6±0.7	75.28±0.5	95.8±0.6	78.98±0.45	75.32±0.63	96.4±0.29	1.04±0.3	1.00±0.2	1.0±0.03
	Petroleum ether	64.2±0.4	74.5±0.5	99.67±0.3	69.23±0.4	71.25±0.65	90.25±0.54	69.98±0.04	73.25±0.5	90.35±0.25	1.05±0.5	1.02±0.2	1.00±0.4
Seed	Ethanol	25.75±0.4	41.75±0.27	56.75±0.2	23.7±0.2	39.6±0.3	51.4±0.2	24.52±0.14	39.2±0.15	51.7±0.2	1.03±0.6	0.98±0.5	1.00±0.4
	Chloroform	62.25±0.34	78.28±0.05	101.5±0.6	60.5±0.3	74.7±0.5	96.3±0.04	60.67±0.4	74.2±0.5	97.2±0.4	1.00±0.45	0.994±.03	0.993±0.5
	Ethyl acetate	63.5±0.7	72.5±0.04	91.8±0.2	62.6±0.7	67.8±0.5	90.8±0.6	61.98±0.5	67.2±0.6	90.4±0.2	0.99±0.36	0.99±0.12	0.98±0.32
	Petroleum ether	64.2±0.4	74.5±0.5	85.68±0.5	69.23±0.4	71.25±0.5	75.5±0.5	69±0.04	71.9±0.5	75.5±0.64	0.99±0.44	1.00±0.2	1.00±0.2

O: Oocytes; M: Microfilarial stage; and A: Adult stage of *Setaria cervi*

^#^Concentrations (μg/ml) of the various solvent extracts of different parts of *C*. *scarabaeoides* plant required to affect 50% of the total population of parasites (Lethal dose (LD_50_) and Inhibitory concentration (IC_50_)) and macrophage cells (RAW 264.7) (Cellular cytotoxicity (CC_50_)) has been provided in the following table. The selectivity index (SI) has also been mentioned in the table from which it is clear that the ethanolic extract of the stem part is superior over other in terms of its antifilarial activity.

**Table 2:** Evaluation of the antifilarial activity of polyphenol-rich ethanolic extract (EECs) of *Cajanus scarabaeoides*.

**EECs**	LD_50_ (μg/ml)			IC_50_ (μg/ml)			CC_50_ (μg/ml)			Selectivity Index		
	O	M	A	O	M	A	O	M	A	O	M	A
	2.5±0.2	10±0.17	35±0.24	1.56±0.16	6.5±0.24	23.67±0.45	2.5±0.27	10.67±0.5	39.79±0.23	1.60±0.54	1.64±0.78	1.68±0.68

O: Oocytes; M: Microfilarial stage; and A: Adult stage of *Setaria cervi*

### EECs exerts potential lethal action against all the developmental stages of *S*. *cervi*

**Fig 2:**
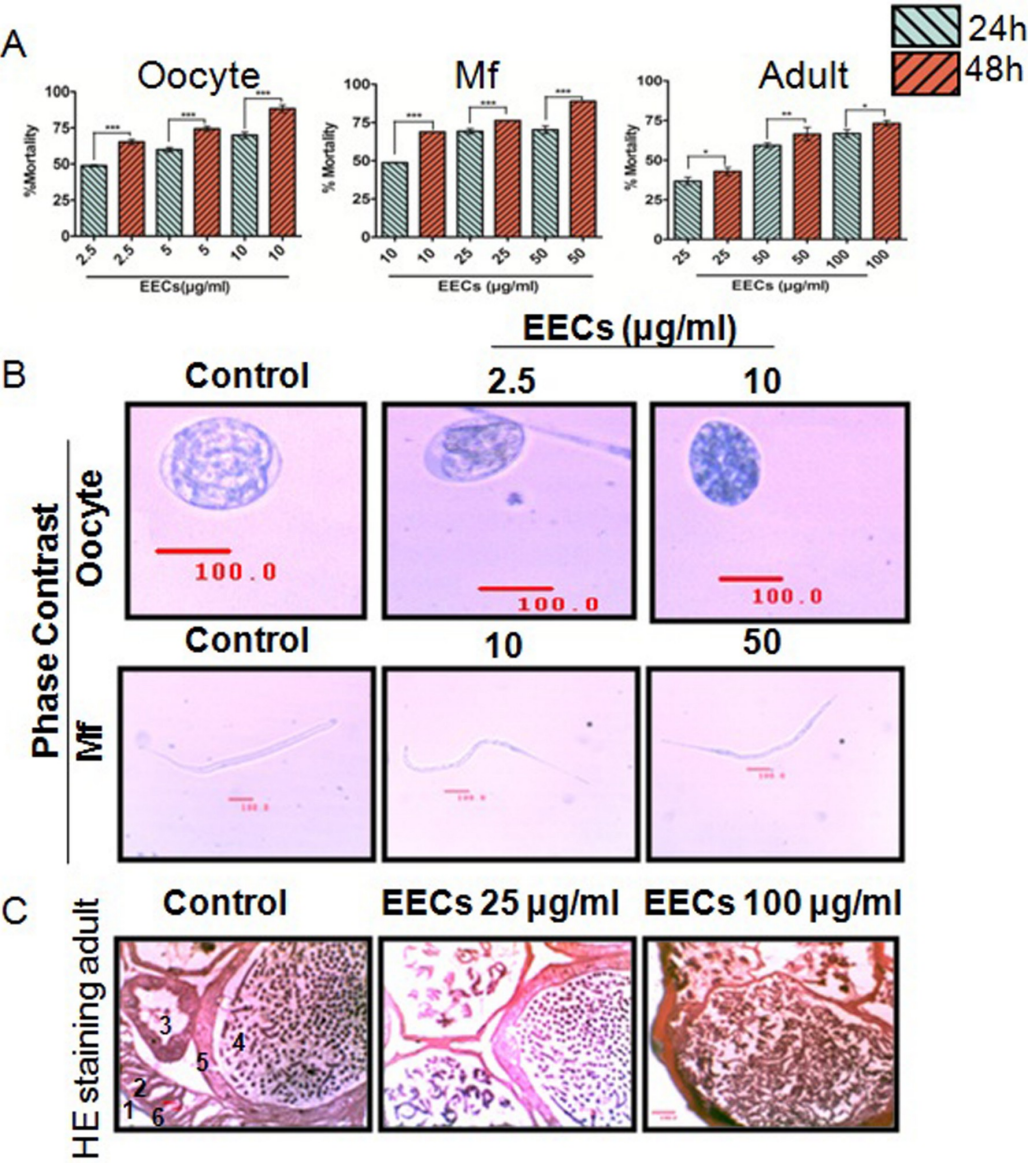
Antifilarial activity of EECs on the oocytes, microfilariae (Mf) and adults of the filarial parasite *Setaria cervi*. **(**A.) Dose-dependent mortality in the oocytes (left panel), Mf (middle panel) and adult stage (right panel) of *S*. *cervi* treated with variable doses of EECs. (B.) phase contrast micrograph showing morphological changes in the EECs treated oocytes (upper panel) and Mf (lower panel). (C.) Histological changes in the adult of *S*. *cervi* after EECs treatment. Control section (i) showed no significant morphological damages in (1) cuticle, (2) longitudinal muscle layer, (3) intestine, (4) uterus, (5) uterine wall, and (6) hypodermis whereas, in (ii) and (iii) shrinkage and morphological alterations in the 25μg/ml and 100 μg/ml of EECs treated parasites were visible at 20X magnification. Scale bar (100 μm) was given using Dewinter Biowizard 4.2 software. Each data is the representative of five independent experiments repeated for at least three times. Data in the bar graph represents mean ±SEM and *p*<0.001 considered as statistically significant.

After optimizing the total polyphenolic content, the active polyphenol-rich extract (EECs) was investigated for its antifilarial potential against the oocytes, Mf and adult stage of the filarial nematode *S*. *cervi*. It was found that EECs can induce mortality on the aforesaid stages of *S*. *cervi* with reasonably low LD_50_ values viz. 2.5μg/ml (for oocytes), 10 μg/ml (for Mf) and 35μg/ml (for adults) (Fig 2).

**Fig 3:**
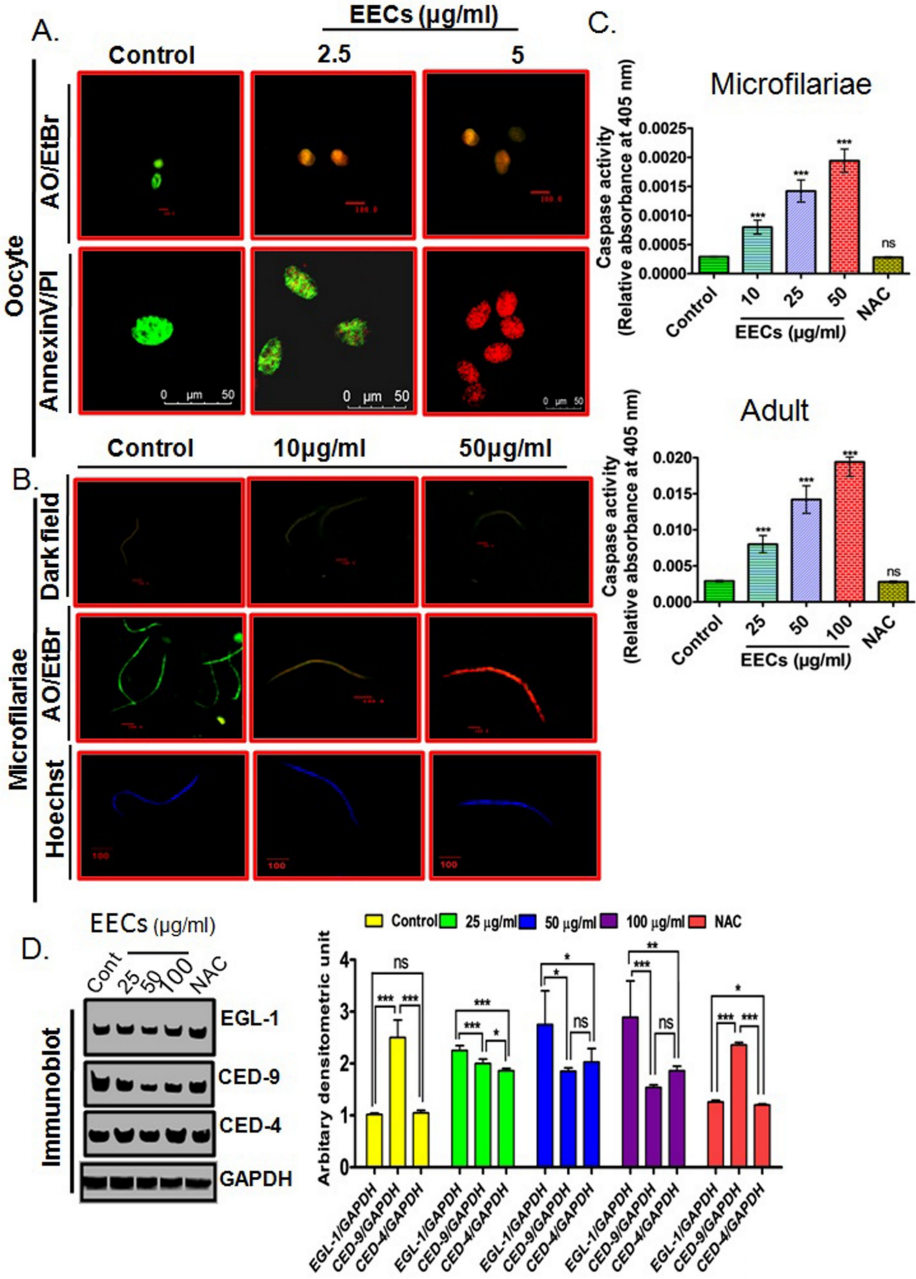
EECs activates apoptotic pathways in *S*. *cervi*. (A.) Annexin V-FITC/PI staining showing outward projection of phosphatidylserine indicating induction of apoptosis in oocytes. (B.) Dark field micrograph (upper panel), AO/EtBr double staining (middle panel) and Hoechst staining (lower panel) depicting the induction of apoptosis in Mf. EECs is efficient in inducing apoptosis in adult *S*. *cervi* and the process is ROS dependent. (C.) Caspase activity in the EECs treated Mf and adults of *S*. *cervi*. (D.) Immunoblots showing expressions of pro- and anti-apoptotic protein in *S*. *cervi* after EECs treatment. Each data is the representative of five independent experiments repeated for at least three times. Data in the bar graph represents mean ±SEM and *p*<0.001 considered as statistically significant.

Induction of mortality in the different developmental stages of *S*.*cervi* was found to be increased in a dose-dependent manner (Fig 2). The effect of EECs was found to be highest at 48 h of treatment *in vitro*, and it showed almost 100% mortality (Table 1). Microscopic investigations of the dead worms revealed shrinkage and membrane damage throughout the body walls of the parasites (Figs 2 and 3) that encouraged us to study the possible molecular mechanism of action of EECs.

DNA fragmentation in EECs treated Mf was evident from our experimental data (Fig 3). Furthermore, Annexin-V-FITC and Propidium Iodide staining of oocytes and acridine orange/ ethidium bromide double staining of both oocytes and Mf further corroborate with the apoptotic activity of EECs (Fig 3).

### EECs induces oxidative damages in *S*. *cervi*

**Fig 4:**
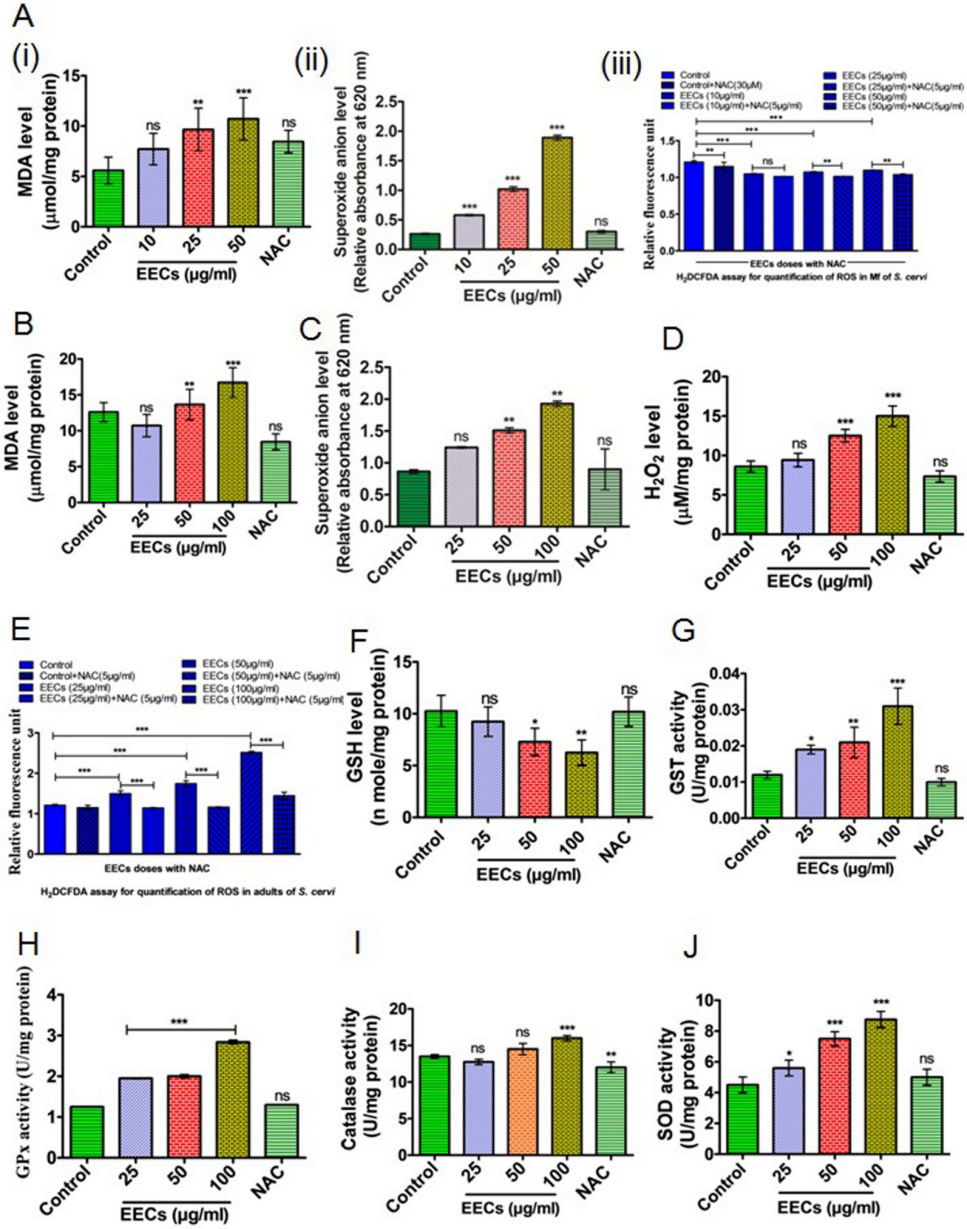
EECs induces oxidative stress in *S*. *cervi* microfilariae (Mf) and adults. (A.). Levels of (i) Malondialdehyde, (ii) Superoxide anion, and (iii) total ROS generated in the microfilarial stage of *S*. *cervi*. (B.) Malondialdehyde, (C.) Superoxide anion, and (D.) H_2_O_2_ in the EECs treated parasites. (E.) Level of total ROS generation in *S*. *cervi* adults after EECs treatment. (F.) Level of reduced glutathione (GSH) in EECs treated parasites. Alteration in the activities of the cellular antioxidant enzymes viz. (G.) Glutathione-S-transferase, (H.) Glutathione peroxidase, (I.) catalase and (J.) SOD in EECs treated *S*. *cervi*. Each data was obtained from the five independent experiments and replicated for at least three times. Data in the bar graph represents mean ±SEM and *p*<0.001 considered as statistically significant.

Our previous investigations on the antifilarial efficacy of phytochemicals showed the involvement of oxidative damages as a major player behind inducing worm mortality [14, 29]. Herein, shrinkage and damage in EECs treated worms (Fig 2C) suggested that there may be an involvement of oxidative damage. We observed an increased level of MDA (an end product of membrane lipid peroxidation) alongside an enhanced generation of superoxide anion and H_2_O_2_ (Fig 4). Moreover, the level of total ROS was significantly (*p*<0.001) increased. Alongside the free radicals, the level of other cellular stress markers i.e. antioxidant enzymes were also upregulated (Fig 4). An increment in the level of catalase, superoxide dismutase, GST and GPx were noticed (Fig 4). Intriguingly, the level of the non-enzymatic antioxidant i.e. GSH was converted. Such a high level of free radicals, enhancements in antifilarial activities and depletion of cellular antioxidants collectively suggested that EECs induced death of *S*. *cervi* is primarily caused by oxidative damages.

### The antifilarial action of EECs is executed by the ROS-activated EGL-1/CED-4/CED-3 pathway of cell death

Induction of oxidative stress in filarial nematode is known to signal activation of apoptotic pathways that actually result in the death of the parasites [5, 15, 16, 20]. Since EECs induces oxidative damages in *S*. *cervi*, therefore it is likely that the apoptotic pathway could be activated by EECs. As postulated, it was observed that EECs treatment results in the activation of EGL-1/CED-4/CED-3 pathway and induction of this pathway is ROS dependent (Fig 4). Expression of the proapoptotic proteins viz. EGL-1, CED-4 were upregulated while expression of the anti-apoptotic protein CED-9 was found to be down-regulated as evident in the immune blots (Fig 4). We have also found that ROS scavenger is capable of utilizing the apoptosis-inducing potential of EECs (Fig 4). We have also found a dose-dependent activation in the caspase activity that corroborates the induction of classical nematode-specific CED pathway leading to effector caspase activation (Fig 2). In the presence of ROS scavenger N-acetyl cysteine (NAC), the phenomena were reversed and indicated that the apoptosis induction was primarily signalled by oxidative stress.

### EECs is nontoxic

**Fig 5:**
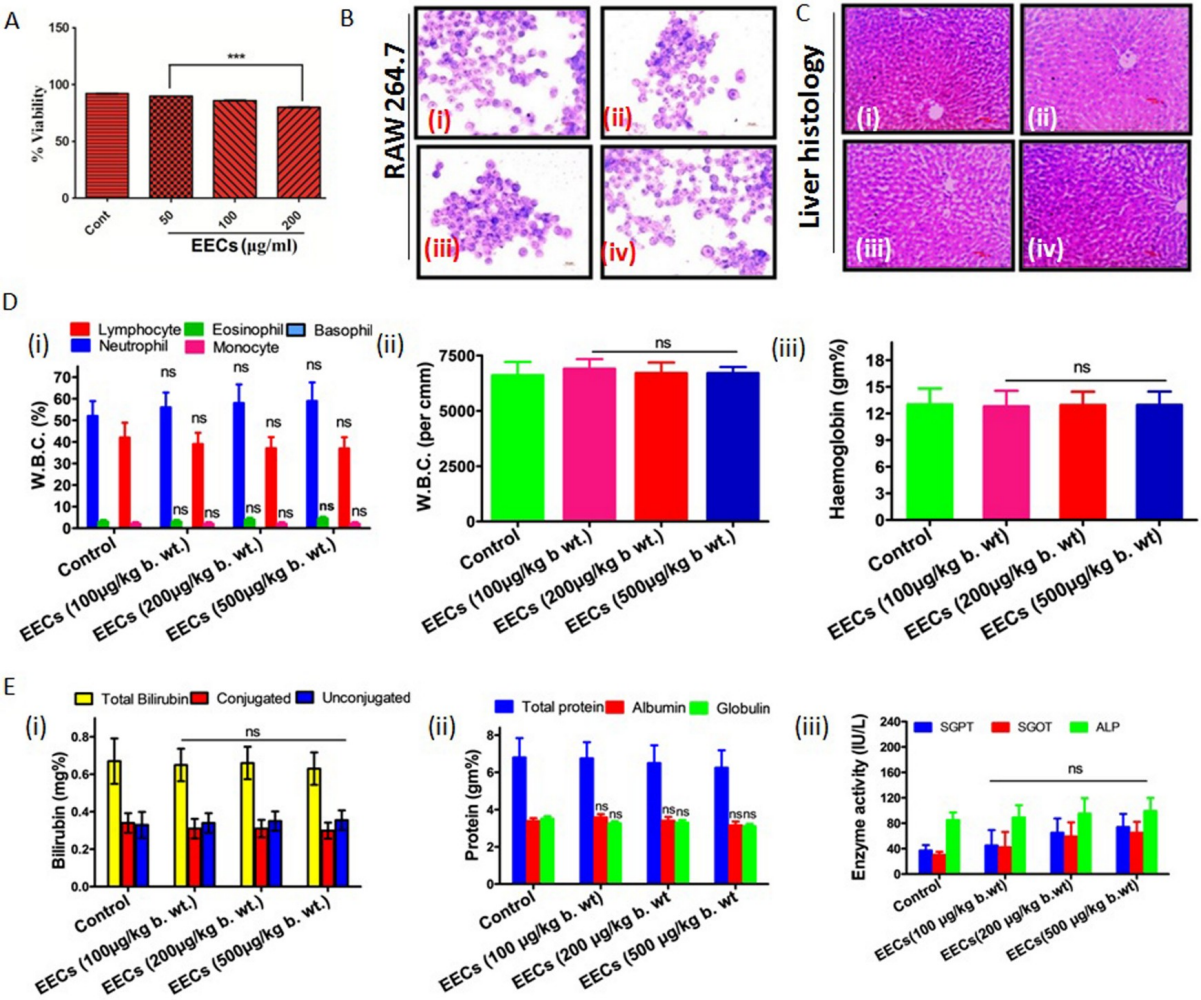
EECs exerts no toxic effect on mammalian cells and tissue. (A.) Viability and (B.) morphology of mouse macrophages (RAW 264.7) treated with EECs. (C.) Histological alteration of liver tissue of Wister rat treated with EECs. (i) represents control; (ii), (iii) and (iv) respectively represent the histological observation of the liver tissue collected from the rats treated with 100, 200 and 500μg/kg body weight. (D.) Alteration in the hematological parameters such as (i) % WBC; (ii) WBC per cubic mm; and (iii) % hemoglobin in the control and EECs treated rats (E.) Alteration in hepatic biomarkers (i) serum bilirubin; (ii) serum proteins and (iii) activities of the serum transaminases (SGPT, SGOT, and ALP) and alkaline phosphates. Each data is a representative of experiments conducted in triplicate and repeated for a further five times.

The major criterion of any natural extract or compound is its benign nature. In our study, we found that EECs is non-toxic as it had not altered the viability of mammalian cells *in vitro* as well as it also not produced any toxic alteration in animal tissues (Fig 5). The *in vitro* studies on the effect of EECs on mouse macrophage cell (RAW 264.7 cell line) revealed no significant cell death up to a dose of 200μg/ml (Fig 5A) and cell morphology was also normal (Fig 5B). Moreover, the selectivity index of EECs was also high that resembles the selective toxicity of EECs on the filarial parasites but not on the mammalian cells (Tables 1 and 2). The *in vivo* toxicity examination demonstrated no detectable alteration in the histology of treated liver sections (Fig 5C). The biomarkers of liver functions viz. SGPT, SGOT, and ALP all were on par with the control (Fig 5E (iii)). In addition, the levels of the hematological parameters like leukocyte count (Fig 5D (i, ii)) and total hemoglobin (Fig 5D(iii)) had also not been altered in EECs treated rats. This evidence is collectively indicative of the nontoxic nature of EECs.

## Discussion

Medicinal plant research is majorly based on the identification and application of the phytoextracts/compounds for treating parasitic infections. Synthetic compounds are preferred in the pharma industry due to several advantages like controlled synthesis, the ability to modify the structure according to the needs and availability [30, 31]. Natural products are preferred for their abundant distribution in nature, wide range of bio-activities, cost-effectiveness in preparing the therapeutic formulation and limited extent of side effects on non-targeted cells and tissues.

Lymphatic filariasis is a debilitating problem among the tropical and subtropical countries to date. Despite the effort of WHO in adopting the control strategy, LF is still taking enormous toll from the human lives with increasing reports of new cases of filariasis worldwide. In this connection, antiflarial drug development is therefore considered as the foremost agenda amongst the filarial researchers. Hitherto, a number of natural, synthetic, semi-synthetic and nanoparticle-based approaches have been emerged out of which most of the compounds failed at the early phase of clinical trial. Thus, the development and/or screening of novel antifilarial therapeutics are the foremost need. Previously, we have shown the antifilarial potential of a number of medicinally important plants viz. *Azadirachta indica* [15], *Nyctanthes arbortristis* [25], *Diospyros peregrina* [26] etc. Herein, we are presenting a new member namely *Cajanus scarabaeoides* to the existing list of the medicinal plants having antifilarial potential.

In the present investigation, we found the potential antifilarial action of *C*. *scarabaeoides* on the filarial nematode *S*. *cervi*. The polyphenol-rich ethanolic extract is effective in killing the filarial parasite in all the developmental stages at a reasonably low dose and the lethal effect is selective only against the parasites but not against the mammalian host. Although we have not isolated the active principle(s) from EECs, our experimental evidence based on HPTLC data confirm that polyphenols are the major bioactive compounds responsible for displaying the antifilarial potential. Previously, we have reported that plant polyphenols like ferulic acid [30], resveratrol [20] and other bioactive phenolics [16] exert potential antifilarial action through induction of ROS leading to programmed cell death. In this study, we have also documented that EECs possesses a very high quantity of resveratrol, ferulic acid as well as other polyphenols, which could be the reason behind such strong antifilarial potential of the plant *C*. *scarabaoeides*.

**Fig 6:**
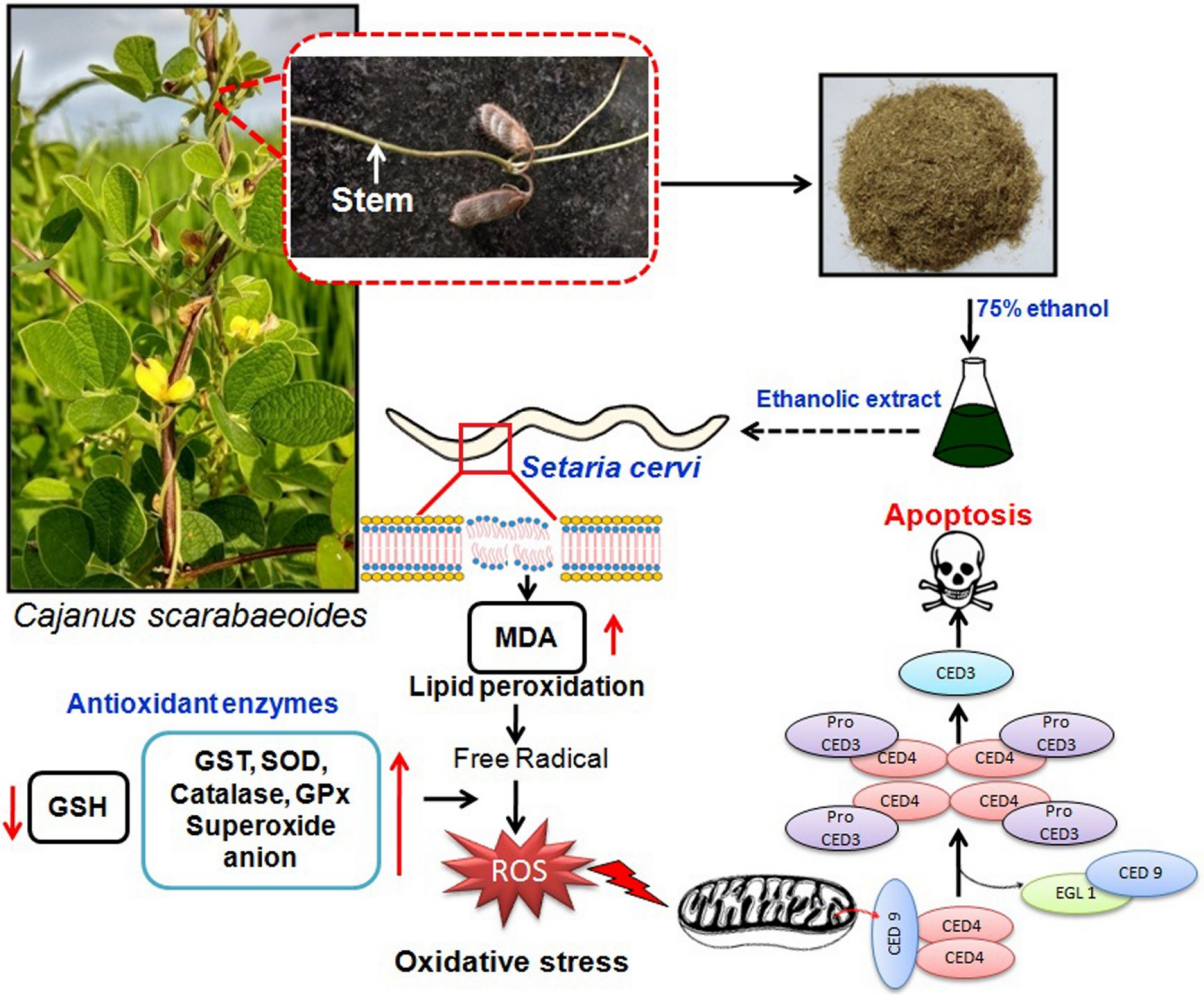
Scheme depicting mechanism of antifilarial activity of EECs.

The strong antifilarial action of EECs on *S*. *cervi* prompted us to study its molecular mode of action. Our experimental data clearly revealed that EECs mainly triggers the oxidative stress by upregulating the pro-oxidants (Superoxide O^-^_2_, H_2_O_2_, MDA) and depleting the antioxidants (GSH, GST, catalase, and SOD). The nematode antioxidant system protects the parasites from the host induced oxidative stress and it is the main reason behind the prolonged survival of the parasites inside the host [32, 33]. The elevation of the level of pro-oxidants is considered detrimental to the parasites as free radical chain reactions damage all sorts of cellular biomolecules [32, 33]. From this investigation it has been clear that EECs increases the production ROS and decreases antioxidant responses- both results together limit the survival of the parasite. Induction of oxidative stress is also coupled to the apoptotic pathway activation [32, 33]. In fact, ROS generated from membrane lipid peroxidation can directly activate CED pathway in the filarial nematode [20, 32, 33]. Herein, an increased level of MDA indicates that EECs potentially induces membrane damage following lipid peroxidation which starts a further chain reaction. As a result, the level of ROS like superoxide, H_2_O_2_, and total ROS is significantly elevated in the parasite tissue after the treatment of EECs. This high pro-oxidative condition is found to signal apoptosis in *S*. *cervi* via activation of the EGL-1/CED-4/CED-3 pathway. The ROS scavenger is found to reverse the pro-oxidative effect of EECs as well as its apoptosis induction potential. This has suggested that EECs induces apoptotic death in *S*. *cervi*, that is primarily signalled from ROS generation. Therefore, the pathway is said to be ROS dependent. The molecular mechanism of antifilarial action of the polyphenol-rich ethanolic extract of *C*. *scarabaoeides* has been presented by a schematic representation (Fig 6).

Last but not least, EECs has been found as absolutely benign with a high CC_50_ value which also indicates towards the future promises of this phytochemical preparation as herbal remedy for treating bancroftian filariasis. Moreover, the copious availability of this plant in the Indian subcontinent is most likely to serve as a cost-effective future phytotherapeutic for the filaria affected individuals.

## Conclusion

In this study, *C*. *scarabaeoides* has shown its tremendous therapeutic potential against all the developmental stages of the filarial parasite *S*. *cervi*. The preparation of the extract that provides ample amount of bioactive phytochemicals is relatively simple, cost-effective, and accurate. The preparation is selectively cytotoxic to the filarial parasite, while is extremely non-toxic to the mammalian cells and tissues. Taken *en masse*, our study is a maiden report on the antifilarial activity of the novel medicinal plant *Cajanus scarabaeoides*. Isolation of the active principle that governs the antifilarial activity of this medicinal plant is currently underway.

## Statistical analysis

All the experiments were conducted in triplicate and repeated for at least five times. Each data represent mean±SD. All the data were subjected to One-way ANOVA analysis followed Tukey’s multiple comparison tests and *p<0*.*05* was considered statistically significant.

## Supporting information

S1 File Analyses of the phytochemicals present in EECs and their relative abundance. (DOC)

S1 Fig Comparative chemo-profiling of EECs and other extracts obtained from *Cajanus scarabaeoides*. (TIF)

S2 Fig HPTLC based chromatogram and corresponding fluorescence spectrogram representing the chemo-profiling of the ethanolic extract obtained from the root, leaf, fruit, and polyphenol-enriched extract of *Cajanus scarabaeoides* stem (ECCs). (TIF)
