# Supplementary material for: Polyphenol enriched ethanolic extract of Cajanus scarabaeoides (L.) Thouars exerts potential antifilarial activity by inducing oxidative stress and programmed cell death
Source: PLoS One. 2018 Dec 6;13(12):e0208201. doi: 10.1371/journal.pone.0208201 (PMC6283587; doi:10.1371/journal.pone.0208201)
Supplement: S1 File — (DOC) [file pone.0208201.s001.doc]

**Supplementary Information**

**Supplementary Figure 1**:

**Comparative chemo-profiling of EECs and other extracts obtained from *Cajanus scarabaeoides*.**

A. Presence of flavonoids in *C. scarabaeoides* extracts alongside EECs based on the standard catechin and quercetin. Loading order Track 1- 200 ng of catechin and quercetin; Track 2- 300 ng of catechin and quercetin; Track 3-400 ng of catechin and quercetin; Track 4- 500 ng of catechin and quercetin; Track 5- 600 ng of catechin and quercetin; Track 6,7,8 Ethanolic extract of *C. scarabaeoides* obtained from root, leaf, fruit and Track 9- Ethanolic extract of *C. scarabaeoides* obtained from stem. B. Presence of phenolics based on the standards galic acid, caffeic acid and ferulic acid. Loading order Track 1- 200 ng of gallic acid, caffeic acid and ferullic acid; Track 2- 300 ng of gallic acid, caffeic acid and ferullic acid; Track 3-400 ng of gallic acid, caffeic acid and ferullic acid; Track 4- 500 ng of gallic acid, caffeic acid and ferullic acid; Track 5- 600 ng of gallic acid, caffeic acid and ferullic acid; Track 6,7,8 Ethanolic extract of *C. scarabaeoides* obtained from root, leaf, fruit and Track 9- Ethanolic extract of *C. scarabaeoides* obtained from stem. Presence of C. resveratrol, Loading order Track 1- 200 ng of resveratrol; Track 2- 300 ng of resveratrol; Track 3-400 ng of resveratrol; Track 4- 500 ng of resveratrol; Track 5- 600 ng of resveratrol; Track 6,7,8 Ethanolic extract of *C. scarabaeoides* obtained from root, leaf, fruit and Track 9- Ethanolic extract of *C. scarabaeoides* obtained from stem; and D. Rutin like compounds in the phytoextract, Loading order Track 1- 200 ng of Rutin; Track 2- 300 ng of rutin; Track 3-400 ng of rutin; Track 4- 500 ng of rutin; Track 5- 600 ng of rutin; Track 6,7,8 Ethanolic extract of *C. scarabaeoides* obtained from root, leaf, fruit and Track 9- Ethanolic extract of *C. scarabaeoides* obtained from stem.

**Supplementary Figure 2**:

**HPTLC based chromatogram and corresponding fluorescence spectrogram representing the chemo-profiling of the ethanolic extract obtained from the root, leaf, fruit and** **polyphenol enriched extract of *Cajanus scarabaeoides*** **stem** (ECCs)**.**

A. HPTLC analysis showing the presence of the flavonoids compounds in the ethanolic stem extract evident in comparison to the reference compounds (catechin and quercentin). Loading order of the samples from the extreme left: Track 1- 200 ng of catechin and quercetin; Track 2- 300 ng of catechin and quercetin; Track 3-400 ng of catechin and quercetin; Track 4- 500 ng of catechin and quercetin; Track 5- 600 ng of catechin and quercetin; Track 6,7,8 Ethanolic extract of *C. scarabaeoides* obtained from root, leaf, fruit and Track 9- Ethanolic extract of *C. scarabaeoides* obtained from stem.

B. HPTLC analysis depicting the presence of the polyphenolic compounds in the ethanolic stem extract. Loading order of the samples from the extreme left: Track 1- 200 ng of gallic acid, caffeic acid and ferullic acid; Track 2- 300 ng of gallic acid, caffeic acid and ferullic acid; Track 3-400 ng of gallic acid, caffeic acid and ferullic acid; Track 4- 500 ng of gallic acid, caffeic acid and ferullic acid; Track 5- 600 ng of gallic acid, caffeic acid and ferullic acid; Track 6,7,8 Ethanolic extract of *C. scarabaeoides* obtained from root, leaf, fruit and Track 9- Ethanolic extract of *C. scarabaeoides* obtained from stem

C. HPTLC analysis showing the presence of the resveratrol like compounds in the ethanolic stem extract. Loading order of the samples from the extreme left: Track 1- 200 ng of resveratrol; Track 2- 300 ng of resveratrol; Track 3-400 ng of resveratrol; Track 4- 500 ng of resveratrol; Track 5- 600 ng of resveratrol; Track 6,7,8 Ethanolic extract of *C. scarabaeoides* obtained from root, leaf, fruit and Track 9- Ethanolic extract of *C. scarabaeoides* obtained from stem

D. HPTLC analysis demonstrating the presence of the rutin like compounds in the ethanolic stem extract. Loading order of the samples from the extreme left: Track 1- 200 ng of Rutin; Track 2- 300 ng of rutin; Track 3-400 ng of rutin; Track 4- 500 ng of rutin; Track 5- 600 ng of rutin; Track 6,7,8 Ethanolic extract of *C. scarabaeoides* obtained from root, leaf, fruit and Track 9- Ethanolic extract of *C. scarabaeoides* obtained from stem
