# Supplementary figures and images for: Polyphenol enriched ethanolic extract of Cajanus scarabaeoides (L.) Thouars exerts potential antifilarial activity by inducing oxidative stress and programmed cell death
Source: PLoS One. 2018 Dec 6;13(12):e0208201. doi: 10.1371/journal.pone.0208201 (PMC6283587; doi:10.1371/journal.pone.0208201)

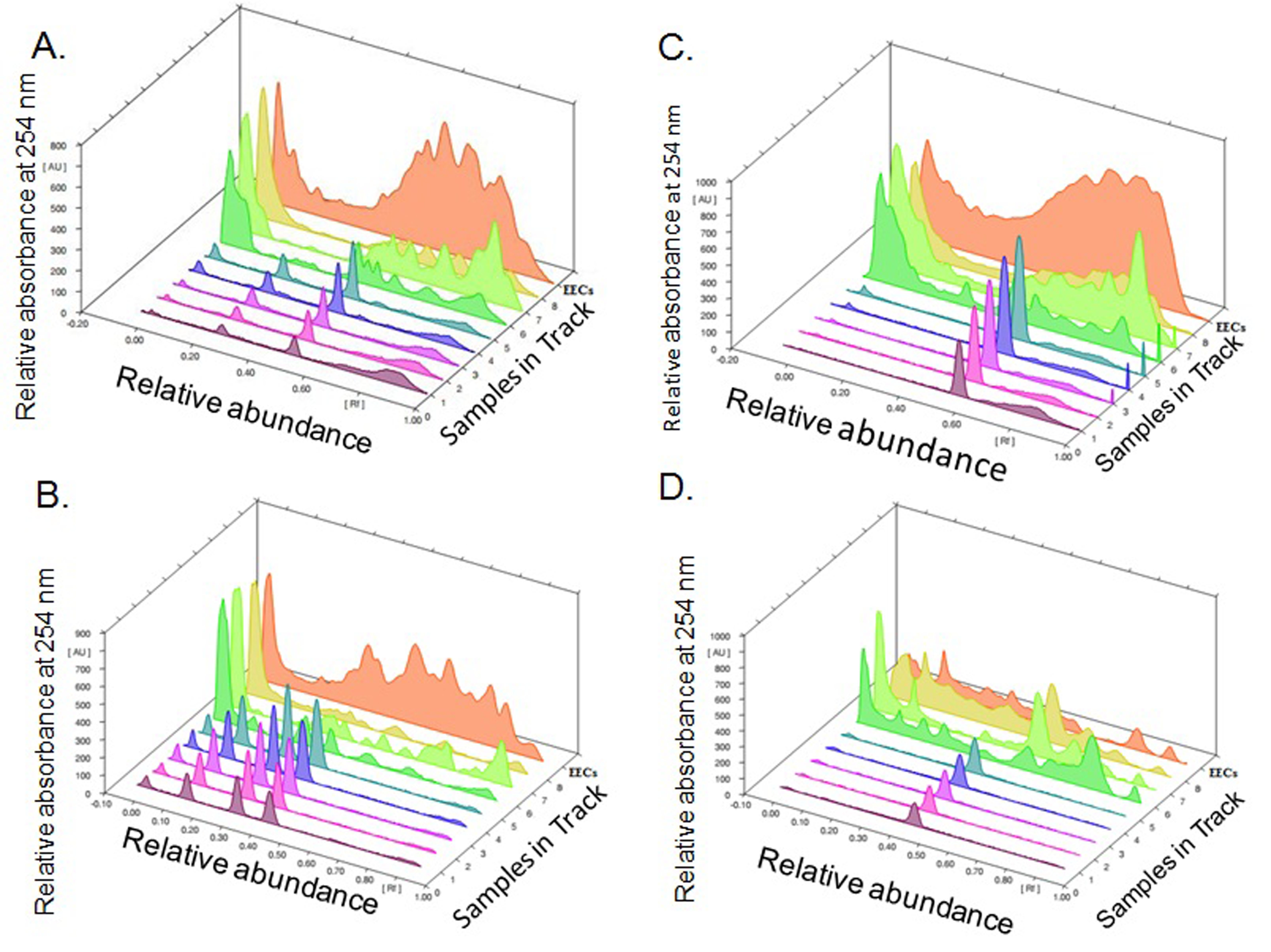

Supplement: S1 Fig — (TIF) [file pone.0208201.s002.tif]

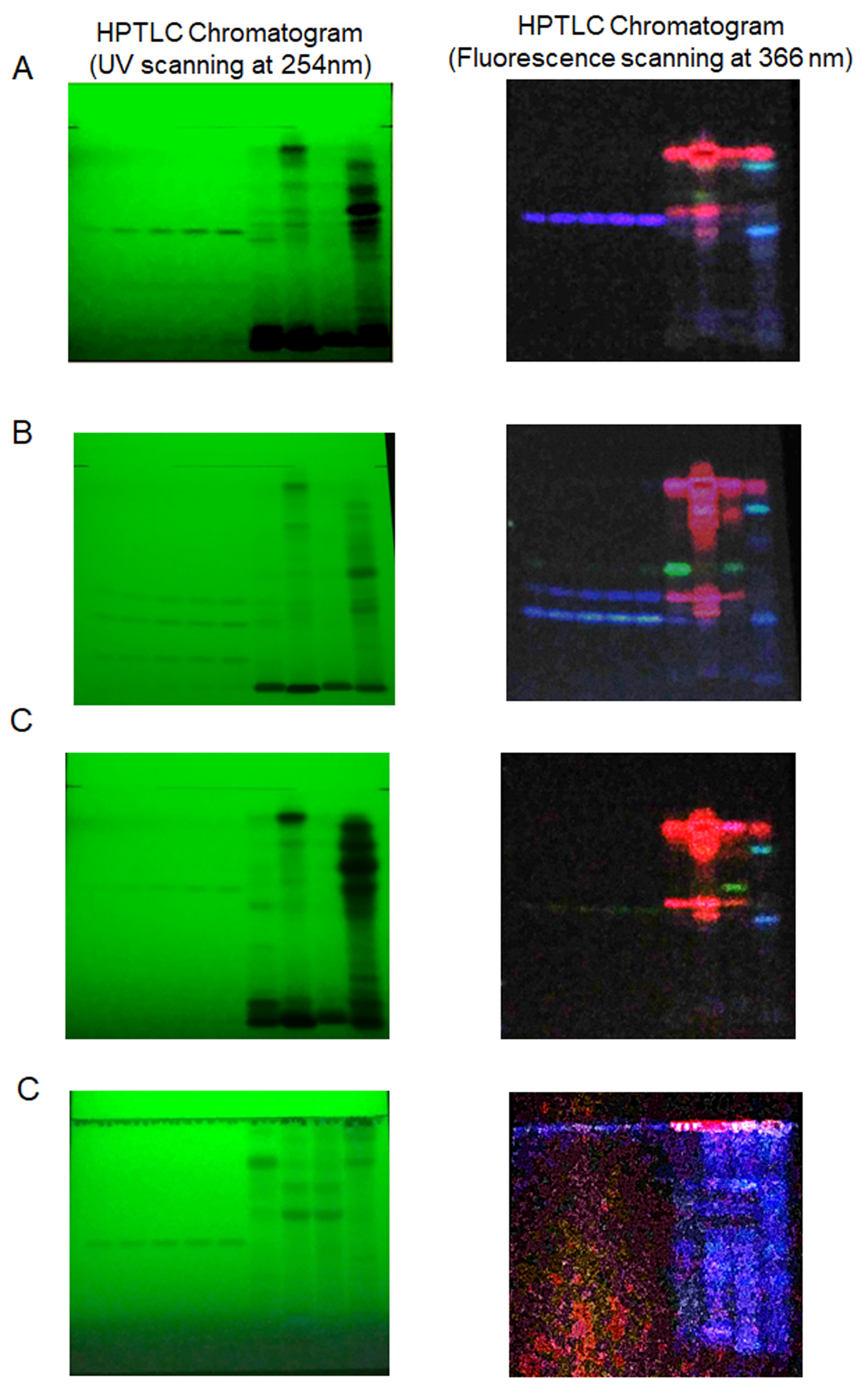

Supplement: S2 Fig — (TIF) [file pone.0208201.s003.tif]
